# “Fiat Justitia” Again

**Published:** 1877-02

**Authors:** 


### Editorial

### "FIAT JUSTITIA" AGAIN.

It is possible we should once more notice the Rome correspondence of the *Dental Cosmos*. When, under the title of "Fiat Justitia," in October, 1876, we kindly corrected a misapprehension, by giving the true history of the Code of Ethics, all of which we knew, and much of which we were, we did so not to enlighten those familiar with the current literature of our profession a dozen years ago, but only that our younger brethren might not be led into error. All thoughtful readers of the journals at the time referred to, know that the Code is, in substance, the one previously adopted by the Ohio State Dental Society at its first meeting, which was prepared by a special committee consisting of Drs. Watt and Taft.

But it is gratifying to know that the Rome correspondent (of the Cosmos, as aforesaid) still believes Dr. Allen "to be the author of the Code of Ethics." Certainly, just according to the fitness of things; for some minds are happier with belief than with knowledge, and are often so full of faith that they have no room for facts, but such are not found among the brightest and best cultivated. The REGISTER would be sorry to disturb such equanimity, and wouldn't try to get the said correspondent to *know* anything—would rather just watch him believe.

About the matter of "Bad Taste," the correspondent claims he had no thought of a personal application, and that the names "Mad River Valley" and "Thunder and Lightning Hills" were "a fortuitous combination." Webster says, "But an event can not be in fact *fortuitous*," which is quite true of this event, at least the correspondent claims to have been "hardly aware that there was such a society as the Mad

River Valley." This concedes the whole matter; for *hardly* aware is aware nevertheless, and he was *fully* aware that there was no "Thunder and Lightning Hills" Society in our profession. The veil is too thin—might as well have been lifted.

Then we are solemnly told that he was never hired to write for dental journals, except as a reporter. And who has said he ever was? The REGISTER intimated nothing of the sort, but kindly cautioned him not to do himself injustice by leading any to suspect him to be identical with a certain "unknown" writer *known* as "Snarler."

The correspondent closes his most recent solemn exercises by coming out at the same end of the *horn* he went in at, in other words, quoting himself thus:—"There have been, and still are, so many dentists who can claim neither finish of education, nor elegance of deportment, that the reproach which they occasion must be borne yet awhile longer, until the harvest of their generation shall be gathered in."

Which is exceedingly sad. Sorrowful, very—very, indeed!

